# Study on structural characteristics, physicochemical properties, and in vitro digestibility of Kudzu‐resistant starch prepared by different methods

**DOI:** 10.1002/fsn3.3079

**Published:** 2022-09-28

**Authors:** Yongmei Guan, Meichen Wang, Xinqi Song, Shenghang Ye, Cheng Jiang, Huanhuan Dong, Weifeng Zhu

**Affiliations:** ^1^ Key Laboratory of Modern Preparation of Traditional Chinese Medicines, Ministry of Education Jiangxi University of Chinese Medicine Nanchang China

**Keywords:** digestive properties, Kudzu starch, physicochemical properties, resistant starch, structural characteristics

## Abstract

Three different methods, including autoclaving, autoclaving–debranching, and purification, were used to prepare Kudzu‐resistant starch (KRS) from Kudzu starch (KS). The physicochemical properties, such as thermodynamic properties, pasting properties, solubility, swelling, and coagulability, as well as the in vitro digestive characteristics of the three kinds of KRS were studied. The results showed that the morphology of starch granules of KRS prepared by autoclave, autoclave enzymatic hydrolysis, and purification methods was changed and the relative crystallinity was significantly decreased compared with the original starch. X‐ray diffraction (XRD) showed that KRS exists in the form of C and C+V crystalline form. There was a significant increase in the pasting temperature and a remarkable decrease in the peak viscosity and the expansion degree of the KRS prepared by all three methods. The solubility of the resistant starch (RS) obtained by autoclaving–debranching and that by purification were both increased compared to that of native KS, while the solubility of the RS obtained by autoclaving was decreased. Meanwhile, the retrogradation of the three RS was also improved to varying degrees. The contents of RS in the samples were: P‐KRS (71%) > DA‐KRS (43%) > A‐KRS (42%) > KS (9%). Simulated human in vitro digestion experiments showed that RS has stronger antidigestibility properties than native starch. Among them, the RS prepared by the purification method has stronger antidigestive properties, and it is predicted that it may have a better potential value in regulating blood glucose. These results indicated that the processing properties of KRS, especially the digestibility, are significantly improved and can be used as a new functional food ingredient, which deserves thorough study.

## Graphical abstract

Three different methods including autoclaving, autoclaving‐debranching, and purification were used to prepare Kudzu resistant starch from Kudzu powder. The physicochemical properties, such as thermodynamic properties, pasting properties, solubility, swelling and coagulability, as well as the in vitro digestive characteristics of the three kinds of kudzu resistant starch were studied.

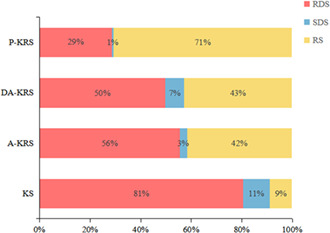

## 1 INTRODUCTION

Kudzu (*Pueraria lobata*) is considered a classic medicinal food homologous plant, widely distributed in the countries of East Asia and America. Kudzu has been reported to have various health‐maintaining pharmacological functions, including antidiabetic activity, antihyperlipidemic activity, and prevention of cardiovascular diseases, due to numerous active components such as isoflavones, saponins, polysaccharides, and starch (Duru et al., 2020; Penetar et al., 2015; Wang, Zhu, et al., 2017; Yang et al., 2016). Kudzu has been widely used in Chinese medicine and food production. Kudzu contains 53% ~ 66% carbohydrate and is mostly in the form of starch. Modern research on *Pueraria lobata* has mainly focused on small‐molecule active ingredients, and large amounts of starch have been discarded after large‐scale extraction, resulting in a waste of resources.

According to different classification methods, starch can be divided into rapidly digestible starch (RDS), slowly digestible starch (SDS), and resistant starch (RS) (Englyst et al., 1992). RS, a product of starch digestion, is poorly absorbed by the small intestine and has a beneficial effect on human health (Englyst & Cummings, 1985). RS has received a lot of attention because of its physiological functions similarly to those of the traditional dietary fiber. Previous studies have shown that RS has many beneficial effects on human health, including the prevention of obesity, diabetes, and colon cancer (Bindels et al., 2017; Birt et al., 2013; Giacco et al., 2014; Zhang et al., 2020).

Researchers have been dedicating their efforts to the preparation of RS because of its ability to offer a broad range of applications. Methods for preparing RS include heat treatment, enzyme treatment, combined heat and enzyme treatment, and chemical treatment. Heat treatment has the advantages of simple operation, low cost, high yield, no pollution, etc. (Sajilata et al., 2006). The enzyme treatment method not only has the above advantages, but also has strong enzyme specificity. Nevertheless, chemical treatment is less used in food because of the poor debranching effect, low yield, and low safety. Ultrasonication is an emerging technology that is efficient, environmentally friendly, safe, and easy to apply, but has high production costs (Huang et al., 2016). Guo et al. prepared Kudzu‐resistant starch (KRS) using α‐amylase and glucoamylase and found that the contents of RSD, SDS, and RS were 5.66%, 25.88%, and 68.46%, respectively, and the crystallinity increased from 34.8% to 43.6% with the increase of enzymatic digestion time (Guo et al., 2016). Feng et al. produced KRS with better water‐holding capacity and transmittance than native starch by debranching and subsequent recrystallization and the content of RS was 45.4% (Zeng et al., 2019).

The KRS prepared by the annealing method by Zhu et al. showed an increase in RS content compared to Kudzu starch (KS) content but still very low (2.51% ~ 8.59%) (Zhu et al., 2018). Some chemical modification methods, such as hydrochloric acid hydrolysis, acid hydrolysis–hydroxypropylation, carboxymethylation, cross‐linking, and esterification, were also applied to prepare KRS, but these methods have poor safety (Chen et al., 2017; Li et al., 2012; Tang et al., 2012; Wang et al., 2010; Zhao et al., 2017). Nevertheless, these methods have problems such as complicated preparation process, poor safety, and low RS content, so we will use a simpler and safer method to prepare KRS. Up till now, limited articles are available on the structural characterization of KRS in different treatments and the relationship between digestion resistibility and structure.

In recent years, the demand for nutritious and healthy food has become more and more urgent with the evolution of dietary structure and disease spectrum. In this study, KRS was prepared from Kudzu by autoclaving, autoclaving–debranching, and purification. The purpose of this study was to compare the structural characteristics, physicochemical properties, and digestive characteristics of KS and KRS to lay the foundation for further studies on the blood glucose regulation function of KRS.

## 2 MATERIALS AND METHODS

### 2.1 Materials

Kudzu starch (KS) was purchased from Nanchang Hang Sheng Ecological Agriculture Co, Xinjian, Jiangxi Province, China. Pancreatic α‐amylase (Sigma‐Aldrich Inc., USA), pullulanase (Beijing Solarbio Science & Technology Co., Ltd., China), high‐temperature‐resistant α‐amylase (Shanghai Aladdin Bio‐Chem Technology Co., Ltd., China), glycosylase (Beijing Solarbio Science & Technology Co., Ltd., China), D(+)‐Glucose (Beijing Solarbio Science & Technology Co., Ltd., China). All the other chemicals were of analytical grade.

### 2.2 Preparation of KRS

Autoclaving Kudzu‐resistant starch (A‐KRS): The KS suspension (12%, w/w, dry‐base starch) was heated at 90°C in a water bath for 30 min. The gelatinized starch paste was heated at 121°C for 30 min, cooled at room temperature for 1 h, and refrigerated at 4°C for 12 h. Finally, the starch obtained was dried to a constant weight at 60°C and ground through a 100‐mesh sieve to obtain the resistant‐enhanced A‐KRS.

Autoclaving–debranching Kudzu‐resistant starch (DA‐KRS): Following the autoclave method, take 100 g of KS and allow to cool at room temperature after the autoclave has been completed. Citrate buffer (0.135 mol/L) was used to adjust the pH to 4.5, followed by the reaction between prulanase (60 U/g) and suspension at 40°C for 4 h. After that, the starch suspension was inactivated at high temperature and cooled to room temperature, then the prulanase was removed by centrifugation at 892*g* for 10 min. Later, the mixture was allowed to age for 24 h under refrigeration, dried at 60°C for 12 h, and then crushed and ground through a 100‐mesh sieve to obtain the DA‐KRS.

Purification Kudzu‐resistant starch (P‐KRS): After autoclaving–debranching, the treated starch was prepared with citric acid buffer (0.135 M, pH = 2). Then, 10 ml of pepsin (400 U/ml) was added to 15% starch milk, stirred in a water bath at 40°C, and adjusted the pH to 7, then 10 ml of high‐temperature‐resistant α‐amylase (400 U/ml) was added, stirred in a water bath at 95°C for 30 min, adjusted the pH to 4.5, and added 0.6 g of glycosylase (100,000 U/g), and stirred in a water bath at 60°C for 1 h. The enzyme was inactivated, and the supernatant was removed after repeated washing with 95% ethanol, stirred in the water bath at 60°C for 1 h. After repeated washing, the supernatant was removed, dried at 60°C for 12 h, crushed, and sieved, and the P‐KRS was obtained.

### 2.3 Surface morphology and granule size

The morphology of the dried starch samples was examined using a Quanta 250 FEG scanning electron microscope (SEM) (FEI Ltd., Oregon, USA) with an accelerating voltage of 15 kV (Li et al., 2018). The dried starch materials were sprayed on circular metal stubs previously covered with double‐sided adhesive and coated with gold prior to SEM examination.

The granule sizes of KS and KRS were analyzed using a laser diffraction particle size analyzer (Mastersizer 2000, Malvern). For laser diffraction analysis, starch granules were suspended in distilled water and stirred. A dry method was used with a particle refractive index of 1.55. Duplicate measurements were carried out for each of the starch preparations. Particle size was obtained and expressed in terms of the mean particle size, D (0.1), D (0.5), D (0.9), D (3, 2), and D (4, 3).

### 2.4 Fourier transform infrared (FT‐IR) spectroscopy

The FT‐IR spectra of KS were obtained with an FT‐IR spectrometer (PerkinElmer, USA) using a transmission mode. The starch sample (2 mg) was blended with potassium bromide (KBr) powder (150 mg) by grinding for 10 min under the infrared lamp and pressed into tablets before measurement. Each spectrum was recorded at a resolution of 4 cm^−1^ and at room temperature with 16 scans. Spectra were corrected and then deconvoluted in the range of 4000 ~ 400 cm^−1^ (Zheng et al., 2016).

### 2.5 X‐ray diffractometry (XRD)

The starch samples were analyzed using X‐ray diffractometer (Bruker Corp, Germany) operating at 40 KV and 40 mA at diffraction angle 2*θ* of 4° to 40° with step intervals of 0.02° at a scanning speed of 4°/min. The XRD data were analyzed by Origin, and the relative crystallinity was calculated by the following formula (Nara & Komiya, 1983):

(1) $$\text{Relative crystallinity}\;\left(\%\right)=\frac{A_{c}}{A_{c}+A_{a}}\times 100\%$$

where *A*_*c*_ and *A*_*a*_ stand for the crystal and amorphous areas, respectively.

### 2.6 Differential scanning calorimetry (DSC)

Thermal properties of KS were investigated with the use of a differential scanning calorimetry (DSC‐7000X, HITACHI, Japan). The dried starch sample was thoroughly mixed with distilled water in a ratio of 1:3. The pans were sealed, equilibrated for 24 h at room temperature, and then heated from 20 to 180°C at the rate of 10°C/min. An empty pan was used as a reference. Onset temperature (*T*_0_), peak temperature (*T*_*p*_), conclusion temperature (*T*_*c*_), and enthalpy values (∆*H*) were measured from the DSC thermogram (Zeng et al., 2016).

### 2.7 Rapid visco‐analyzer (RVA)

The pasting properties of KS were analyzed using the RVA‐Techmaster Visco‐Analyzer (RVA‐4) (Perten Instruments, Sweden). Add 25 ml of distilled water into starch samples (3 g of total weight), then increase the temperature from 50°C to 95°C, and the whole procedure takes 13 min until the end (Zheng et al., 2020).

### 2.8 Solubility and swelling power

Starch suspension (1%) was heated at 55°C, 65°C, 75°C, 85°C, and 95°C in a water bath for 30 min and then cooled to room temperature. The suspension was centrifuged at 3000*g* for 10 min (TGL‐200MC, Hunan Xiangxi Instrument Co., Ltd., China). The supernatant and the sediment were separated, an aliquot of the supernatant was evaporated at 110°C, and the swollen starch sediment was weighed (Li et al., 2020). Solubility and swelling power were calculated as follows:

(2) $$S\%=\frac{A}{B}\times 100\%$$

(3) $$P\%=\frac{B}{W\times \left(1-S\right)}\times 100\%$$

where *S* is the solubility power, *A* is the weight of dried supernatant (g), *W* is dried starch weight (g), *P* is the swelling power, and *B* is the weight of sediment (g).

### 2.9 Condensation

Starch suspension (1%) was heated at 95°C for 30 min in a water bath, cooled to room temperature, and then poured it into a graduated cylinder and let it settle. Carefully observe the retrogradation of different starch samples by the ratio of the volume of starch paste supernatant to the total suspension at different times (Yang et al., 2021).

### 2.10 In vitro starch digestibility

The in vitro starch digestibility was determined according to the literature, with minor modifications (Englyst et al., 1996). Briefly, 200 mg of starch sample was precisely weighed and dispersed in 0.1 mol/L sodium acetate solution, the system temperature was maintained at 37°C after homogenization. Then 2.5 ml of α‐amylase (580 U/ml) solution and 2.5 ml of glycosylase (30 U/ml) solution were added. The enzymatic hydrolysate (0.2 ml) at 20 and 120 min was removed and boiled for 5 min to deactivate the enzymes, and then centrifuged at 4000*g* for 10 min. The glucose content in the supernatant was analyzed by the dinitrosalicylic acid (DNS) (DNS reagents were prepared according to National Standard GB T23 874‐2009 and used after seven days) method as mentioned above. Finally, the percentages of RDS, SDS, and RS were analyzed by the following equations:

(4) $$\mathrm{RSD}\%=G_{20}\times 0.9/\mathrm{TW}\times 100\%$$

(5) $$\mathrm{SDS}\%=\left(G_{120}-G_{20}\right)\times 0.9/\mathrm{TW}\times 100\%$$

(6) $$\mathrm{RS}\%=\left(100-G_{120}-G_{20}\right)\times 100\%$$

where *G*_20_ and *G*_120_ indicate the glucose content (mg) released after 20 and 120 min, respectively, and *TW* is the total starch weight (mg).

### 2.11 Kinetics of in vitro enzymatic hydrolysis of starch

Goñi et al. (1997a) method was adopted with slight improvements. Draw the hydrolysis curve of different starch samples at 10, 20, 30, 40, 60, 90, 120, 180 min, and perform first‐order kinetic fitting of the hydrolysis curve (Li et al., 2018; Shi & Gao, 2011). The formula is as follows:

(7) $$C=C_{\infty }\times \left(1-e^{-\mathrm{kt}}\right)$$

The area under the hydrolysis curve (AUC) was calculated using the following equation:

(8) $$\mathrm{AUC}=C_{\infty }\left(t_{f}-t_{0}\right)-\left(C_{\infty }/k\right)\left\{1-\exp\left[-k\left(t_{f}-t_{0}\right)\right]\right\}$$

where *C*_*∞*_ is the equilibrium concentration (%), *k* is the reaction kinetic constant (min^−1^), *t*_*f*_ is reaction termination time (180 min), and *t*_0_ is initial reaction time (0 min).

The area of white bread under the hydrolysis curve (0 ~ 180 min) was used as the standard, and the HI (%) was calculated as the percentage of the integral area of the hydrolysis curve released from the sample compared to that of white bread. The GI (%) of the samples was estimated as follows:

(9) GI=39.71+0.549×HI

### 2.12 Statistical analyses

All analyses were carried out in triplicate, and the values were described as the means ± standard deviations. Data were subjected to the analysis of variance (ANOVA) by SPSS, and the significance level was determined at the 95% confidence level (CI) (*p* < 0.05).

## 3 RESULTS AND DISCUSSION

### 3.1 Surface morphology and granule size

**FIGURE 1:**
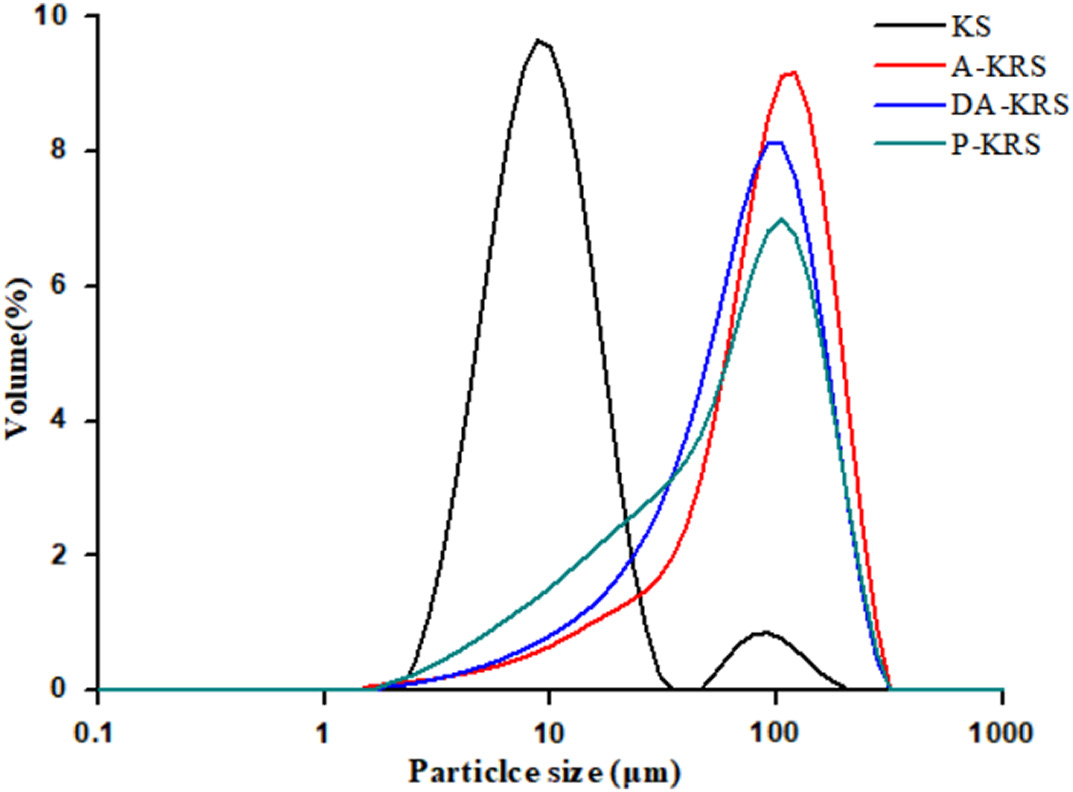
Particle size distribution of different starch samples

The average particle size of native KS was small, with d (0.5) of 8.54 ± 0.09 μm and d (0.9) of 17.95 ± 0.90 μm, as shown in Table 1. The particle size distribution of different starch samples is shown in Figure 1. According to the results, the particle size of native KS appears as two peaks, indicating that the particle size distribution is not uniform and there are obvious particle size differences. The three kinds of KRS have a larger particle size range and particle size than the native KS, and the volume distribution is complete, showing an obvious unimodal curve, indicating that part of the KS particles swelled, aggregated, or crystallized repeatedly during the process of forming the RS. In addition, the single peak of A‐KRS is sharper than that of DA‐KRS. The uneven particle size distribution of P‐KRS indicates that the granule texture is tight after multiple enzymatic hydrolyses, and the degree of recrystallization is reduced.

**TABLE 1:** Particle size distribution of different starch samples ($\bar{X}$ ± S, *n* = 3)

Sample	d (0.1)/μm	d (0.5)/μm	d (0.9)/μm	D (3,2)/μm	D (4,3)/μm
KS	4.32 ± 0.08^a^	8.54 ± 0.09^a^	17.95 ± 0.90^a^	7.61 ± 0.02^a^	13.21 ± 0.44^a^
A‐KRS	22.60 ± 0.53^b^	87.79 ± 0.20^b^	170.75 ± 0.64^b^	41.39 ± 0.69^b^	93.83 ± 0.37^b^
DA‐KRS	18.13 ± 0.28^c^	72.63 ± 0.65^c^	152.22 ± 0.45^c^	36.43 ± 0.40^c^	80.19 ± 0.32^c^
P‐KRS	10.64 ± 0.26^d^	64.48 ± 1.08^d^	151.66 ± 1.17^c^	25.87 ± 0.38^d^	73.63 ± 0.82^d^

*Note*: Results are expressed as mean ± standard deviation. d (0.1), d (0.5), and d (0.9) are the particle diameters with volume fractions of 10%, 50%, and 90%, respectively. D (3,2) is the average particle size of the surface area, and D (4,3) is the volume average particle size. Values followed by different superscripts in the same column are significantly different (*p* < 0.05).

### 3.2 Granule morphology

**FIGURE 2:**
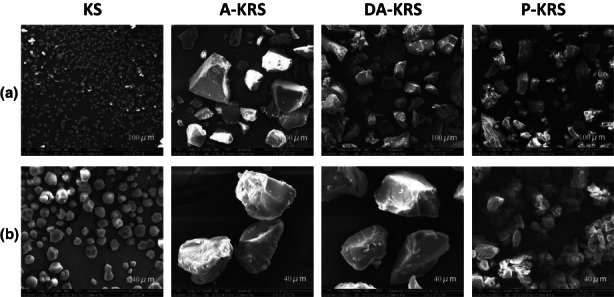
Scanning electron microscope (SEM) images of different starch samples. (a) shows magnification values ×800 and (b) indicates magnification values ×3000.

The shape and surface characteristics of KS, A‐KRS, DA‐KRS, and P‐KRS starches are shown in Figure 2. The KS particles have an irregular spherical‐like structure, smooth and round surface, and small particle size. The A‐KRS particles collapsed, and their volume increased several times. It may be related to the fact that the amylose releasing at high temperature returns to the double helix state and aggregates into crystals during the aging process, forming a more compact structure, and thus the surface shows a rough granular appearance with eroded surface. In the case of DA‐KRS, because of the enzymatic action of prulanase, the amylopectin molecules were shed and the short linear molecules were aged and recrystallized, resulting in a more compact starch structure, thus showing interlaced microporous channels and grooves on the surface. The P‐KRS was processed by amylase and glucoamylase, the soluble components were completely hydrolyzed, which made the insoluble RS particles more compact. Therefore, it can be speculated that the swelling and recrystallization of the amorphous regions of starch may lead to its resistance to enzymatic digestion and fermentation in the large intestine (Zhang et al., 2014).

### 3.3 FT‐IR analysis

**FIGURE 3:**
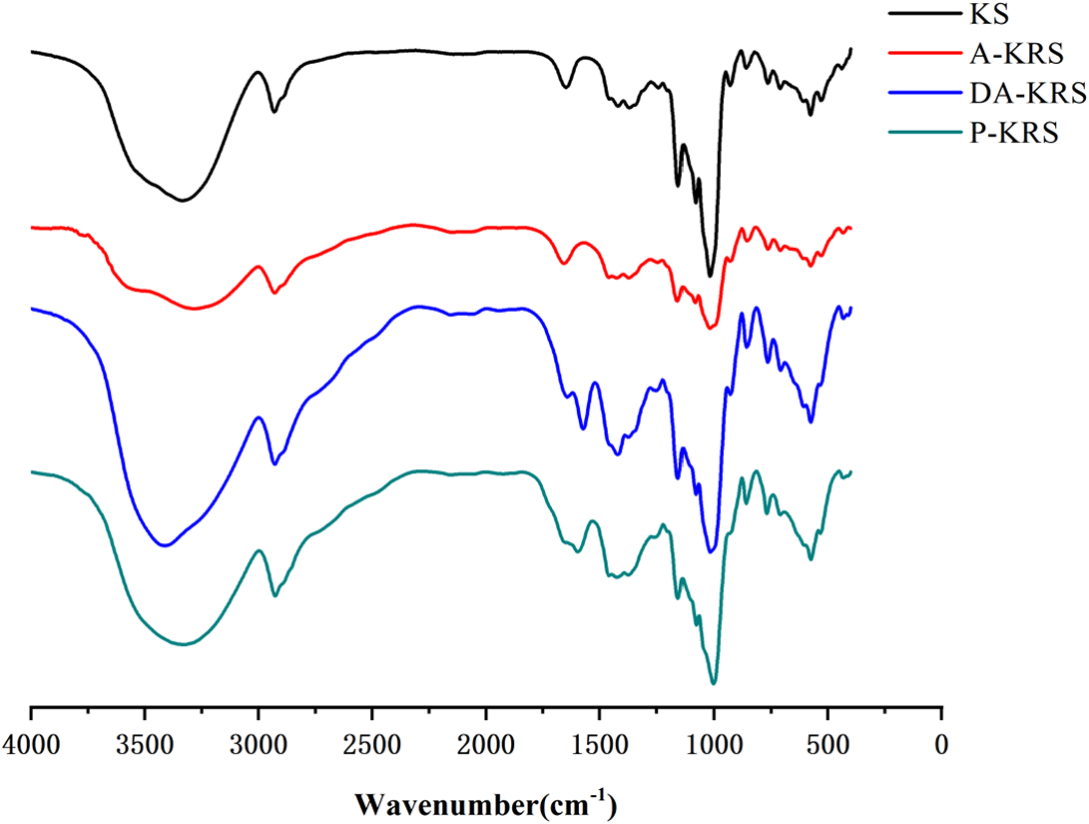
Infrared spectra of different starch samples

Infrared spectroscopy was used to detect changes in functional groups during the formation of RS. As shown in Figure 3, in the absorption band of 3100 ~ 3700 cm^−1^, the absorption curves of the three kinds of KRS were significantly different from that of KS. Due to the high‐pressure treatment during the preparation process, the amylose in the starch granules spilled, and the intermolecular hydrogen bonds weakened, so that the absorption strength of the A‐KRS was significantly reduced compared with the native KS. As to the DA‐KRS and P‐KRS, the process of enzymatic hydrolysis resulted in the precipitation of short‐chain molecules which reassembled then into a compact double helix structure with significantly enhanced amplitude. The three kinds of RS showed different vibration absorption peaks in the fingerprint area compared to KS, which may be related to the hydrogen bond association of RS in water (Taguet et al., 2014). The band at 1750 cm^−1^ was attributed to –C=O stretching vibration (Wang et al., 2019). The heat treatment did not cause chain breakage, so there was no change in the absorption intensity of A‐KRS at 1750 cm^−1^. After enzymatic treatment, the double helix structures of DA‐KRS and P‐KRS become more compact, and therefore the absorption is enhanced. Further FTIR spectrum analyses of starch revealed that the absorption band at 2750 cm^−1^ was generated by the C–H stretching vibration (van Soest et al., 1995). The partial hydrogen bond breakage of DA‐KRS and P‐KRS in the presence of prulanase and α‐amylase resulted in a shift at 2750 cm^−1^.

The ratio of the intensity of bands at 1047 cm^−1^ and 1022 cm^−1^ expresses the degree of short‐range order which was influenced by the changes in band position and band shape. (van Soest et al., 1995). And the bands at 1047 cm^−1^ and 1022 cm^−1^ are associated with the ordered and amorphous structures of starch, respectively (CAPRON et al., 2007). Of the starches in this study, the 1047 cm^−1^/1022 cm^−1^ ratio followed the order: KS (1.048) > DA‐KRS (1.046) > P‐KRS (1.044) > A‐KRS (1.016). Debranching and circulating temperature crystallization changed the short‐range structure, resulting in a higher degree of native starch order, as evidenced by a decrease in the 1047 cm^−1^/1022 cm^−1^ ratio of DA‐KRS and P‐KRS, and the results were agreement with those of Zeng et al. (2019). A‐KRS is only processed by the temperature cycling, so the 1047 cm^−1^/1022 cm^−1^ ratio is larger than those of DA‐KRS and P‐KRS.

The ratio of absorption peak intensity at 995 cm^−1^/1022 cm^−1^ can be used to reflect the changes in the internal double helix structure of starch granules (Wang, Li, et al., 2017). Moreover, the absorption at 995 cm^−1^ is correlated with the crystallization region (van Soest et al., 1995). Some studies have shown that branched starch chains interact with each other to form a lattice structure, linking the double helix structure and forming a crystalline region, while straight‐chain starches form an amorphous region (Valenzuela‐Lagarda et al., 2021). According to the infrared spectrum, the ratios of absorption peak intensity at 995 cm^−1^/1022 cm^−1^ of KS, A‐KRS, DA‐KRS, and PA‐KRS were 1.031, 1.003, 0.998, and 0.974. DA‐KRS was enzymatically cleaved by prulanase, leading to the breakage of α‐1,6 glycosidic bonds and a decrease in amylopectin content, while P‐KRS was further cleaved by α‐amylase, breaking the α‐1,4 glycosidic bonds of both branched and straight‐chain amylose, leading to a further decrease in amylopectin content. Therefore, the 995 cm^−1^/1022 cm^−1^ P‐KRS was lower than that of DA‐KRS. The double helix structure of A‐KRS suffered some degree of damage at high temperatures, while KS was not damaged by high temperatures and enzymes and consequently had the most complete double helix structure.

### 3.4 Crystalline structure

**FIGURE 4:**
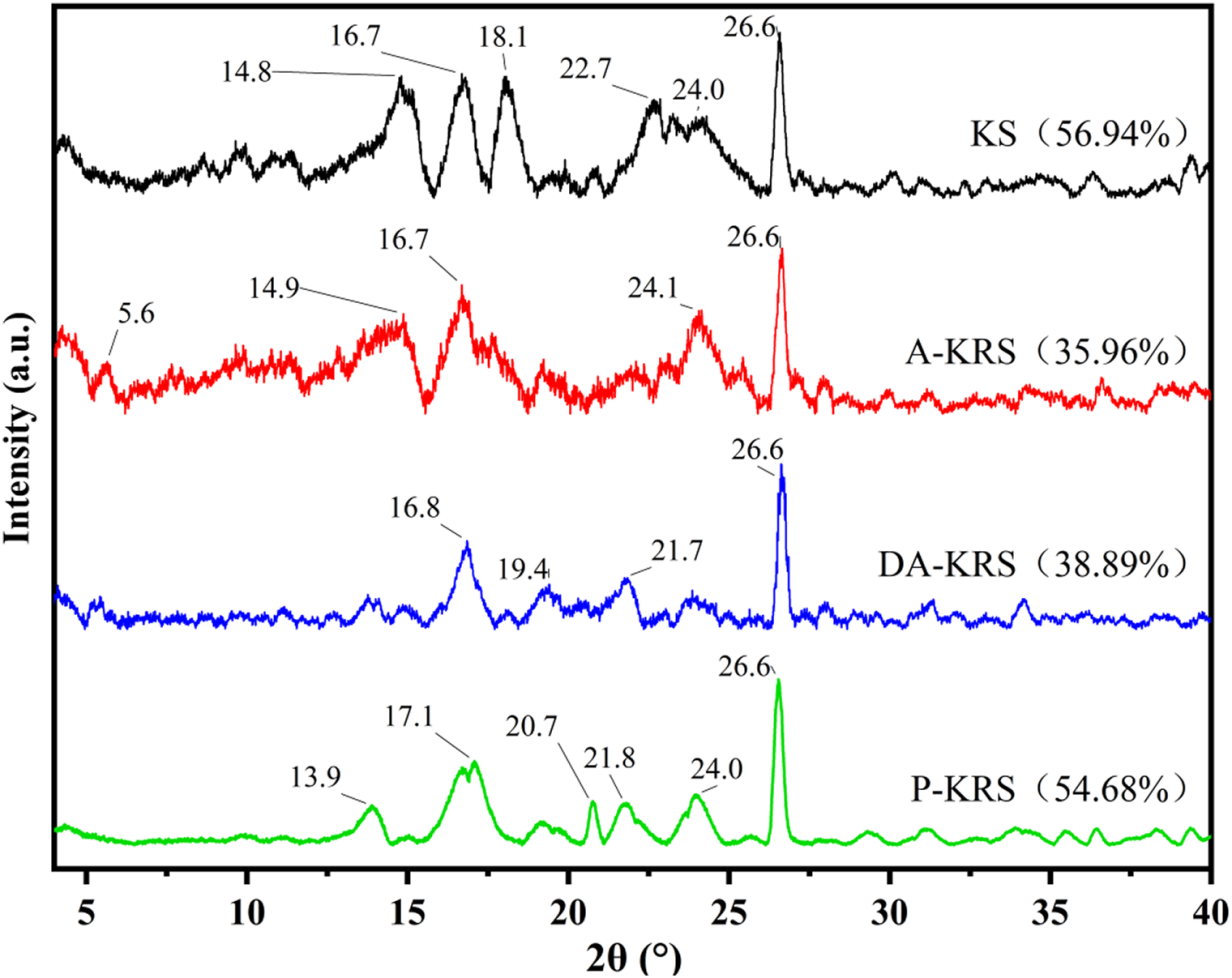
Crystalline structure of different starch samples

The XRD patterns and relative crystallinity of KS, A‐KRS, DA‐KRS, and P‐KRS are presented in Figure 4. All samples exhibited a strong peak at 26.6°, which is typically type A crystal peak (Ma et al., 2018). C type crystallite is a combination of A and B type polymorphs. The KS showed the typical C type diffraction pattern with peaks at 14.8°, 16.7°, 18.1°, 22.7°, 24.0°, and 26.6°. Most of the articles suggested that KS is C type (Wang, Chen, et al., 2017). A‐KRS also displayed a typical C type crystal structure, with characteristic peaks at 5.6°, 14.9°, 16.7°, 24.1°, and 26.6°. DA‐KRS showed the same crystal pattern as A‐KRS, and the diffraction peaks at 16.8°, 19.4°, 21.7°, and 26.6°. After purification treatment, a significant change happened to P‐KRS with reflection intensities at 2θ angle values of 13.9°, 17.1°, 20.7°, 21.8°, 24.0°, and 26.6°, which indicated the transformation from C type to C+V type. Previous studies have shown that the debranched starch shows a B+V complex crystalline structure (Zeng et al., 2019). The variation of crystallinity is influenced by the crystal size of starch, the amount of crystallization, the orientation of the double helix, and the degree of interaction between the double helices (Song & Jane, 2000). The relative crystallinity followed the order: KS > P‐KRS > DA‐KRS > A‐KRS. During the thermal regeneration of starch molecules, some of the molecular chains remain free, which results in a lower crystallinity of RS than KS. DA‐KRS formed new crystals after debranching by prulanase, so the crystallinity was higher than that of A‐KRS. The increased crystallinity of P‐KRS was due to the hydrolysis of the amorphous layers of starch by the action of high‐temperature‐resistant α‐amylase, and the amorphous more crystalline peak decreased slightly, thus the crystallinity increased (Cai & Wei, 2013), and the results were consistent with the results reported by Guo et al. (2016).

### 3.5 Thermal properties

Starch gelatinization is a process of energy transfer, and the thermodynamic parameters of different starches have obvious differences. The thermodynamic parameters of different starch samples are listed in Table 2. Compared with KS, To, Tp, and Tc of the three resistant starches were increased to variable degrees, and the increase in DA‐KRS and P‐KRS was particularly more significant than A‐KRS, which may be related to the formation of new crystals in the starch after enzymatic hydrolysis. Higher temperatures are required to change the stable crystal structure of starch. Thus, To, Tp, and Tc of A‐KRS were higher than those of KS, however, the significant differences were smaller. The starch molecules only undergo molecular chain opening and closing during the high‐pressure treatment process, without debranching or chain cleavage, thus it is difficult for the A‐KRS to form crystals and mostly exists in the form of free starch chains. RS is a tightly spiral structure formed by the agglomeration of amylose, making it more stable than the native starch. Therefore, the phase change requires a higher temperature than the native starch, which is expressed as a higher energy. The enthalpy change (ΔH) of DA‐KRS is higher than A‐KRS because of the formation of new crystals by DA‐KRS. The ΔH of P‐KRS was significantly higher than that of the other two types of RS. This may be because the treatment of amylase and glucoamylase during the preparation of P‐KRS removed the soluble substances and made the crystal structure of P‐KRS more compact. This compact structure requires more energy for the qualitative change and thus P‐KRS exhibits a higher enthalpy change (Lin et al., 2014). The thermal stability of KRS made by the hydrochloric acid modification method used by Li et al. was poorer than that of KS, while the KRS made by our method had better thermal stability (Li et al., 2012).

**TABLE 2:** Thermodynamic parameters of different starch samples ($\bar{X}$ ± S, *n* = 3)

Sample	*T*_0_ (°C)	*T*_*p*_ (°C)	*T*_*c*_ (°C)	*T*_*c*_–*T*_0_ (°C)	Δ*H* (J/g)
KS	47.95 ± 3.53^a^	66.12 ± 8.78^a^	85.28 ± 15.73^a^	37.33 ± 14.72^a^	55.47 ± 11.07^a^
A‐KRS	53.79 ± 2.86^b^	85.94 ± 1.10^b^	98.66 ± 3.38^b^	44.87 ± 1.11^a^	362.20 ± 44.99^b^
DA‐KRS	54.05 ± 1.89^b^	95.54 ± 2.88^c^	106.34 ± 2.14^b^	52.29 ± 3.60^b^	559.63 ± 76.05^c^
P‐KRS	57.70 ± 4.69^b^	96.63 ± 3.19^c^	107.33 ± 1.61^b^	49.63 ± 5.22^a^	668.71 ± 50.17^d^

*Note*: Values followed by different superscripts in the same column are significantly different (*p* < 0.01). (*T*_0_) is the onset temperature, (*T*_*p*_) is the peak temperature, (*T*_*c*_) is the conclusion temperature, (*T*_*c*_–*T*_0_) is the temperature change range, and (Δ*H*) is the enthalpy change.

### 3.6 Pasting properties

There were different changes in the starch granules of KRS prepared by different processing processes, such as heating and enzymatic hydrolysis. Whether the starch swells during heating or maintains its stable structure after enzymatic hydrolysis has a considerable influence on its viscosity after gelatinization (Jay‐lin, 2006). The pasting properties of starches analyzed using RVA are summarized in Table 3. KRS granules prepared by different methods were all more thermally stable, and all the gelatinization indexes were reduced compared to the native KS after heating into paste. DA‐KRS and P‐KRS had a much lower viscosity characteristic index than KS and were unable to gel below 100°C. This may be due to the fact that DA‐KRS and P‐KRS form a more compact crystal structure with small intermolecular gaps, which prevents the powder from punching and absorbing water to form a paste at the set temperature. Compared with DA‐KRS and P‐KRS prepared by enzymatic digestion, the A‐KRS prepared by pressure and heat treatment exhibited higher pasting characteristics, and some characteristics, such as Valley and Final viscosities, were close to those of the original starch. This may be related to the fact that autoclaving acts mainly on the amorphous regions of the starch. During this high pressure and temperature process, the A‐KRS starch molecules absorbed water and formed stable hydrogen bonds for adsorption, resulting in a significant increase in intermolecular gravitational forces, which directly leads to higher viscosities (Govindasamy et al., 1996). The addition of 5% RS to the noodles reduced the viscoelasticity of the noodles, which facilitated less chewing, increased satiety, and reduced carbohydrate intake, but had little effect on the cooking characteristics of the noodles (Punia et al., 2019). Raungrusmee et al. developed gluten‐free noodles with low glycemic index using rice RS, while the addition of xanthan gum improved the texture of RS gluten‐free noodles (Raungrusmee et al., 2020). Therefore, adding the right amount of RS to pasta products can make the performance and flavor of pasta products better and better meet the needs of consumers.

**TABLE 3:** Rapid visco‐analyzer (RVA) profile characteristics of different starch samples ($\bar{X}$ ± S, *n* = 3)

Sample	Peak viscosity (cP)	Valley viscosity (cP)	Breakdown viscosity (cP)	Final viscosity (cP)	Setback viscosity (cP)	Pasting temperature (°C)	Peak time (min)
KS	4577.0 ± 21.3^a^	2816.3 ± 55.1^a^	1760.7 ± 66.9^a^	3952.7 ± 36.7^a^	1136.3 ± 33.4^a^	77.3 ± 0.4^a^	5.2 ± 0.1^a^
A‐KRS	2704.7 ± 54.6^b^	2642.0 ± 41.5^a^	62.7 ± 15.5^b^	3533.0 ± 68.5^a^	891.0 ± 27.1^b^	71.5 ± 0.5^b^	6.2 ± 0.1^b^
DA‐KRS	29.3 ± 1.5^c^	28.3 ± 1.5^b^	1.0 ± 0.0^c^	51.0 ± 1.7^b^	22.7 ± 0.6^c^	–	–
P‐KRS	10.3 ± 0.6^d^	5.0 ± 0.0^c^	5.3 ± 0.6^c^	8.3 ± 0.6^c^	3.3 ± 0.6^d^	–	–

*Note*: Values followed by different superscripts in the same column are significantly different (*p* < 0.01). (–) means the data were not measured.

### 3.7 Swelling power and solubility

**FIGURE 5:**
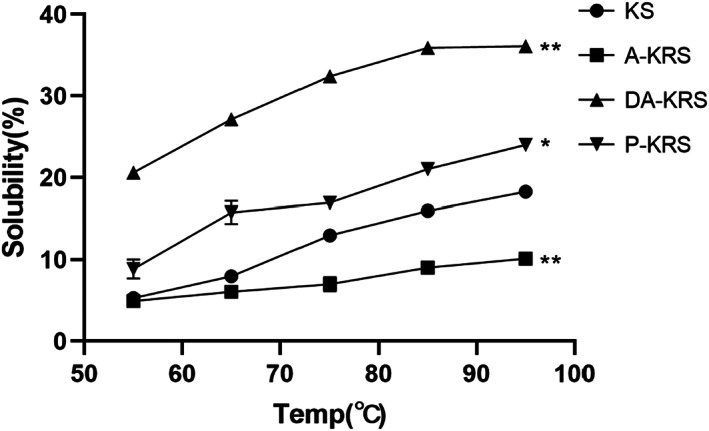
Solubility of different starch samples. (*) shows a significant difference between Kudzu‐resistant starch (KRS) and Kudzu starch (KS) (*p < 0.05, **p < 0.01).

**FIGURE 6:**
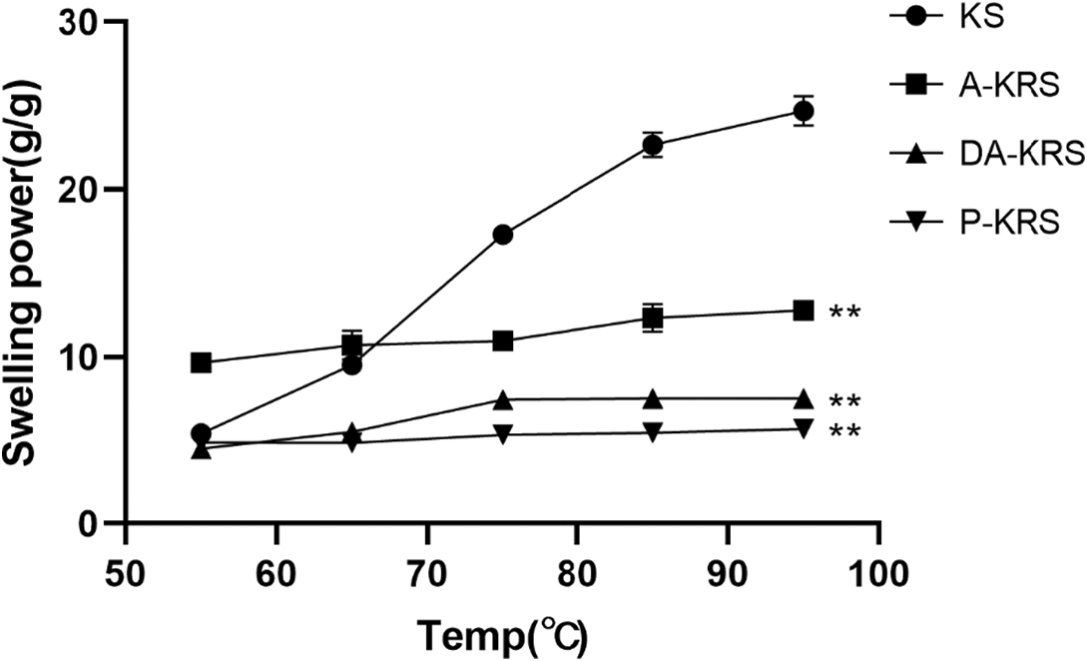
Swelling power of different starch samples. (*) shows a significant difference between Kudzu‐resistant starch (KRS) and Kudzu starch (KS) (*p < 0.05, **p < 0.01).

Swelling power and solubility behavior of KS, A‐KRS, DA‐KRS, and P‐KRS starches at different temperatures are shown in Figures 5 and 6, respectively. The solubility and swelling degree of starch are the key indicators for judging the hydrogen bond force between starch and water (Chen et al., 2015). As shown in Figure 5, with the increase of solution temperature, the water solubility of each starch sample was enhanced significantly, which was consistent with the dissolution behavior of starch in water. Overall, the RS obtained by compression heat treatment exhibited poor solubility. In contrast, the solubility of RS prepared by enzymatic treatment was significantly enhanced compared with the native starch. In particular, DA‐KRS had superior solubility properties, suggesting its better potential for application in food processing. The lower water solubility of A‐KRS may be related to its stable crystal structure, which provides better encapsulation of small molecules of starch. Moreover, the stable crystal structure makes it difficult for the starch to absorb water, which is manifested by the difficulty of swelling effect, as shown in Figure 6. While DA‐KRS and P‐KRS had undergone enzymatic treatment, the structure of the microcrystalline bundle of starch molecules was loosened, and the starch molecules could easily escape with water under high‐temperature conditions (Barua et al., 2021; Fang et al., 2021). P‐KRS was purified after enzymatic digestion, and most of the soluble sugars and small molecules were removed, resulting in higher crystallinity but significantly lower solubility and swelling power.

### 3.8 Condensation

**FIGURE 7:**
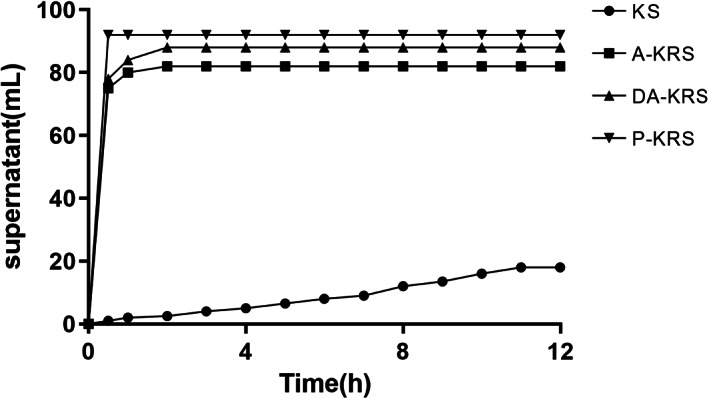
Supernatant of different starch samples

The condensation of starch reflects the ability of starch molecules to hydrogen bond with water (Biduski et al., 2018). As shown in Figure 7, the supernatant volume of samples followed the order: P‐KRS > DA‐KRS > A‐KRS > KS. The starch molecule of native KS had a strong ability to bind with water because it had not been processed by high‐temperature metamorphosis, thus the structural features were more significant after binding with water, as well as its gel density after gelation was close to that of water. Accordingly, the precipitation speed is slower, appearing as a smaller supernatant volume, as shown in Figure 7. The molecules of A‐KRS, DA‐KRS, and P‐KRS were highly helical and had the structural characteristics of aged starch, and this structure was difficult to be destroyed when reheated. RS has low water‐holding capacity and is suitable for use as a quality improver in low‐moisture foods, such as bread, cookies, cakes, etc. Cross‐linked wheat RS with low water‐holding capacity was added to the cookies to make the dough softer and to produce cookies with high fiber and low glycemic index, and the width and thickness values of the cookies were significantly increased and the quality was significantly improved (Kahraman et al., 2019). The addition of RS to solid beverages and jams can enhance the stability and textural properties and keep them unstratified for a long time (Gao et al., 2021).

### 3.9 In vitro starch digestibility

**FIGURE 8:**
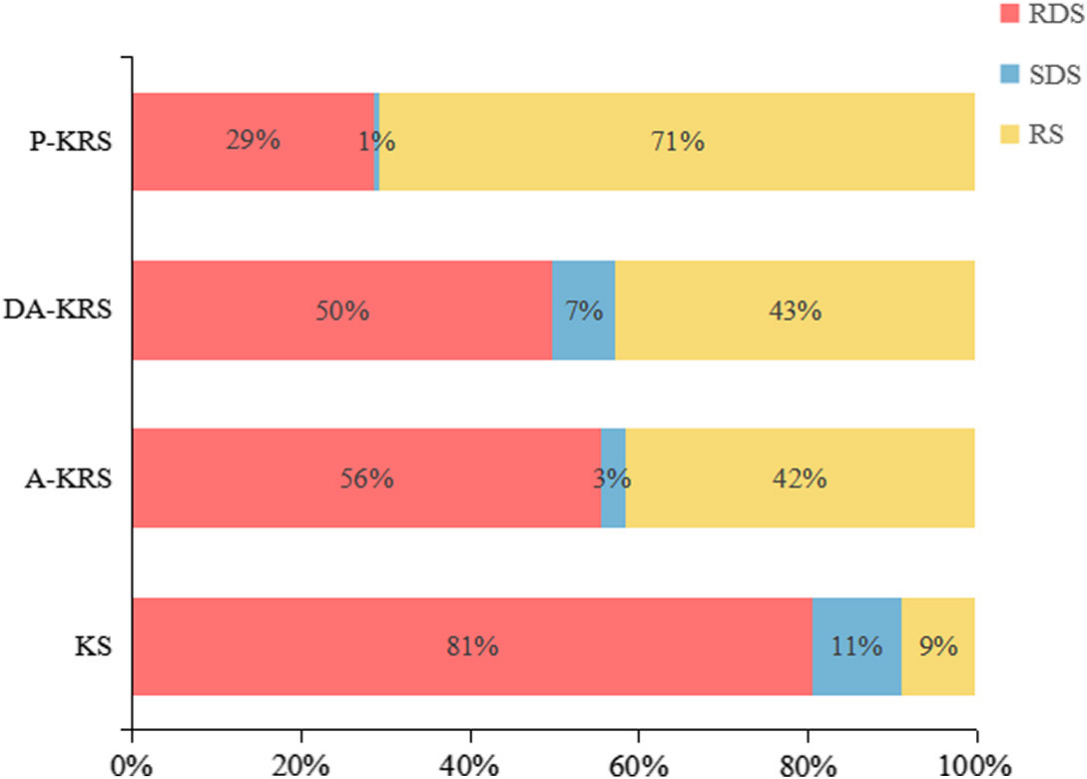
Digestion characteristics of different starch samples

Digestion of starch granules is a complex process, which is influenced by the surface characteristics and internal structure of the starch granules and the starch crystal structure, as well as the interaction of many factors, such as starch source, straight‐chain starch content, and relative crystallinity (Ambigaipalan et al., 2011). RDS, SDS, and RS contents of KS, A‐KRS, DA‐KRS, and P‐KRS are presented in Figure 8. KS was contained in 81% RDS, 11% SDS, and 9% RS, but the high content of RDS leads to a high glycemic index, which is harmful to health. After autoclaving, the content of RDS decreased while RS increased significantly. This change might be caused by the reduction of enzyme contact sites due to the destruction of starch crystal structure. The different starch ratios of P‐KRS followed the order: 71% RS > 29% RDS > 1% SDS. The higher RS content in P‐KRS was attributed to short amylose molecule that recrystallizes to form a dense structure. Amylase has removed excess small molecules of sugar, thus increasing the RS content. As a functional food, RS has been given great importance benefit for decreasing the incidence of enteric disease. Moreover, RS is a good substrate for butyrate production, which can alter microRNA (miRNA) levels in colorectal cancer cells to reducing risk associated with a high red meat diet (Humphreys et al., 2014). In addition, a great number of RS is used as food additive, thereby giving favorable brittleness and tenderness to bread crumbs (Djurle et al., 2018).

### 3.10 Kinetics of in vitro enzymatic hydrolysis

**FIGURE 9:**
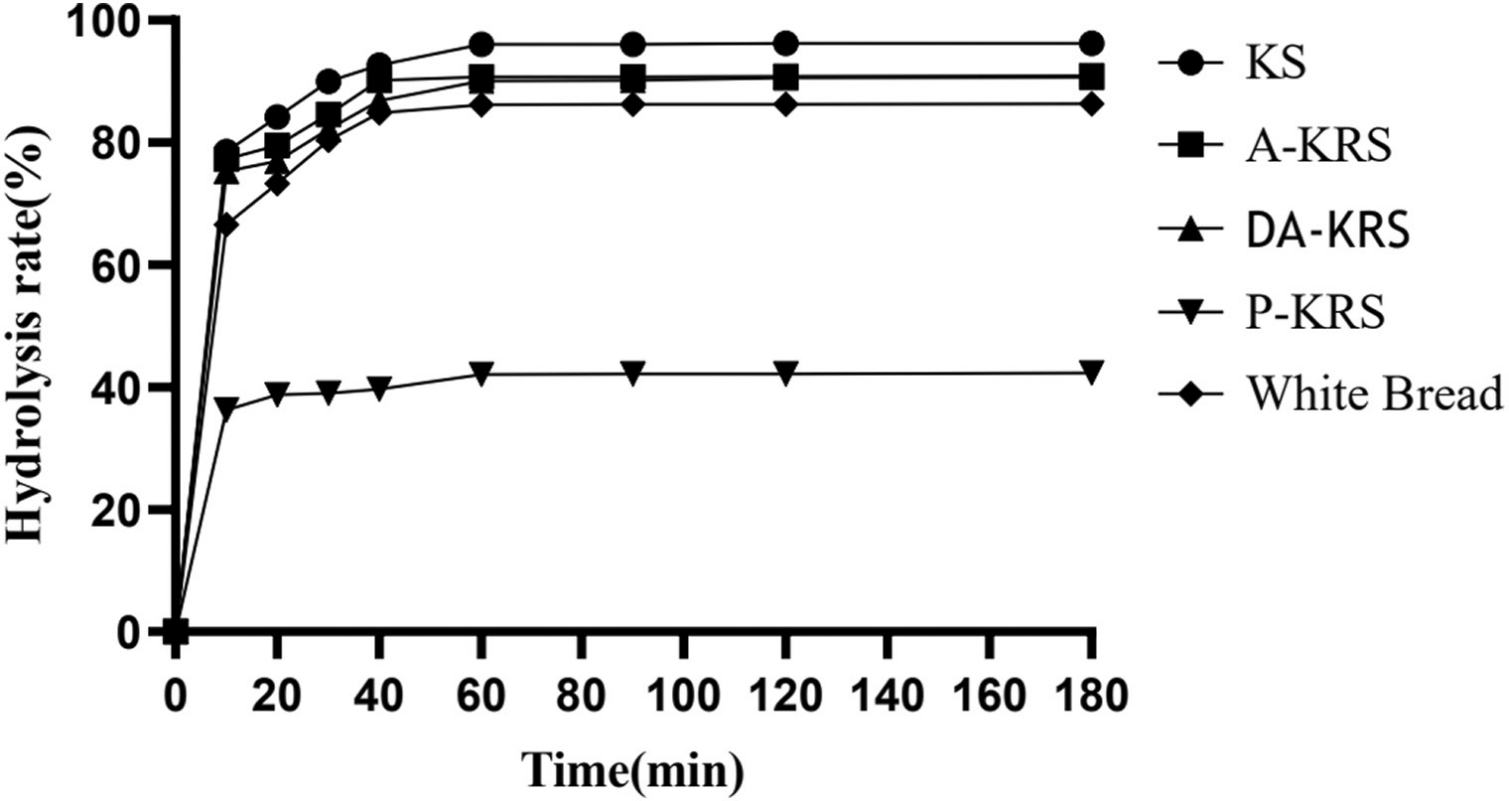
Digestive kinetic curve of different starch samples

The in vitro enzymatic hydrolysis rates of KS, A‐KRS, DA‐KRS, and P‐KRS are shown in Figure 9. The trends in hydrolysis rates for the types of starch and white bread controls were relatively similar. The hydrolysis rate was fast during the first 10 min at the beginning of digestion, and after 10 min, the degree of hydrolysis increased slowly and the hydrolysis rate decreased significantly, 60 min later, the starch hydrolysis was completely completed and the total hydrolysis rate remained stable. According to the hydrolysis curve of starch samples, the digestion process of degradable starch could be divided into two stages: rapid hydrolysis and uniform hydrolysis. The degree of hydrolysis of KS and three kinds of KRS was basically consistent with the content and change trend of RDS, SDS, and RS measured by the Englyst method. KS contains 81% RDS, so the digestibility is high. The surface of A‐KRS starch granules is layered, and DA‐KRS starch granules have pores, thus it is easy for α‐amylase adsorption and enzymatic digestion (Dhital et al., 2010). The lowest digestibility of P‐KRS was attributed to the complexation of starch with lipids to form V‐shaped crystals, which inhibited the water absorption and swelling of starch granules and reduced the binding sites between starch and enzyme molecules, resulting in enzyme resistance (Crowe et al., 2000). The short interval of starch digestion kinetics monitoring can accurately reflect the changes in the middle and late stages, and can more accurately quantify the degree of digestion of each part of starch. The digestion rate may be more suitable for practical situations (Goñi et al., 1997b).

The digestive kinetic parameter of different starch samples is listed in Table 4. The equilibrium concentration of the samples is quite different, and the *C*_∞_, HI, and GI of native starch are the highest. A‐KRS and DA‐KRS had lower digestive kinetic parameters compared to KS, while purified P‐KRS had the lowest HI and GI. This may be due to the high RS content of P‐KRS of 70%, which makes the starch resistant to digestion. The *k* and *C*_∞_ of most starch samples showed the same change trend, the digestible starch content was high, and the value of *C*_∞_ and the speed of reaching the maximum equilibrium concentration increased correspondingly. The *C*_∞_ in KS is 0.94, which is much larger than that of KRS, but its *k* value is only 0.16, indicating that the hydrolysis of KS may be a process from fast to slow or uniform. Then according to the content of SDS in KS, the increase of SDS content has a direct impact, while the increase of RS content can significantly reduce *C*_∞_, HI, and GI (Jiang et al., 2013).

**TABLE 4:** Digestive kinetic parameter of different starch samples ($\bar{X}$ ± S, *n* = 3)

Sample	*C*_∞_ (%)	*k* (min^−1^)	*R* ^2^	AUC	HI	GI
KS	0.94 ± 0.01^a^	0.16 ± 0.02^a^	0.98983	164.17	111.78	101.08
A‐KRS	0.89 ± 0.01^b^	0.18 ± 0.03^a^	0.98714	155.72	106.03	97.92
DA‐KRS	0.88 ± 0.02^b^	0.17 ± 0.03^a^	0.98103	153.96	104.83	97.26
P‐KRS	0.41 ± 0.01^c^	0.20 ± 0.03^a^	0.99084	72.17	49.14	66.69
White bread	0.85 ± 0.01^d^	0.14 ± 0.01^b^	0.98952	146.87	100.00	94.61

*Note*: Values followed by different superscripts in the same column are significantly different (*p* < 0.01). (*C*_∞_) is the equilibrium concentration, (*k*) is the reaction kinetic constant, (AUC) is the area under the hydrolysis curve, (GI) is the glycemic index, and (HI) is the hydrolysis index.

## 4 CONCLUSION

This study showed that the structural characteristics, physicochemical properties, and in vitro digestive characteristics of A‐KRS, DA‐KRS, and P‐KRS were different from those of KS. The KS, A‐KRS, DA‐KRS, and P‐KRS had similar FT‐IR spectra but have differences in absorption intensity indicating different crystallites and double helical structures. Furthermore, the KS, A‐KRS, and DA‐KRS all displayed typical C type crystallization; however, P‐KRS exhibited a complex C+V type crystallization, due to the formation of starch–lipid complexes after enzymatic debranching and recrystallization. The thermal properties and condensation of KS were lower than those of KRS. Among the three KRS, the thermal properties and condensation of P‐KRS were the highest, followed by DA‐KRS and then A‐KRS. On the contrary, the pasting properties and swelling power were in the order of KS > A‐KRS > DA‐KRS > P‐KRS. Compared to KS, KRS had higher RS content but lower RDS and SDS, and the ranking of RS content was P‐KRS > DA‐KRS > A‐KRS > KS. The preparation of KRS by the autoclaving method is simple and fast, and that by autoclaving–debranching is safer, while the starch prepared by the purification method has a high content of RS. The present study recommends that the purification of KS might be a better treatment for producing high RS, with improved pasting properties, better thermal stability, low swelling power, and low glycemic index. Also, the information obtained from this study can be used by manufacturers and researchers to produce Kudzu‐containing products.

## AUTHOR CONTRIBUTIONS

Conceptualization, Y.G, M.W., X.S., H.D., and W.Z.; methodology, Y.G., X.S., and H.D.; data curation, Y.G. and X.S.; writing–original draft preparation, Y.G., X.S., and M.W.; writing–review and editing, Y.G., H.D., and M.W.; visualization, S.Y. and C.J.; supervision, Y.G., H.D., and W.Z. All authors have read and agreed to the published version of the manuscript.

## CONFLICT OF INTEREST

The authors declare no conflict of interest.

## Data Availability

All datasets presented in this study are included in the article.
